# Near-Infrared Spectroscopy and Machine Learning for Geographic-Origin Screening of *Dendrobium crepidatum* Lindl. et Paxt.

**DOI:** 10.3390/foods15142416

**Published:** 2026-07-08

**Authors:** Yingying Hu, Jiecai Li, Guona Dai, Meng Cui, Ying Zhou, Yongcheng Yang, Conglong Xia, Ying Wang, Baozhong Duan

**Affiliations:** 1 School of Pharmacy, Dali University, Dali 671003, China; 13170760928@163.com (Y.H.); 18387263993@163.com (J.L.); 15241821063@163.com (Y.Y.); long7484@126.com (C.X.); 2 International Joint Laboratory for the Development and Utilization of Traditional Chinese Medicine Resources in Yunnan Province, Dali 671003, China; xd202209@126.com (G.D.); cmeng99@126.com (M.C.); 3 Institute of Dendrobium in Longling County, Baoshan 678300, China; zhy929@126.com; 4 Yunnan International Joint Laboratory of Characteristic Medicinal and Edible Resources, Dali 671003, China

**Keywords:** *Dendrobium crepidatum* Lindl. et Paxt., near-infrared spectroscopy, machine learning, geographic origin discrimination, spectral variable interpretation

## Abstract

*Dendrobium crepidatum* Lindl. et Paxt. is a medicinal *Dendrobium* species whose quality and market value may vary with geographic origin, making rapid origin traceability important for batch management, market supervision, and application promotion. This study used near-infrared spectroscopy (NIRS) combined with multivariate analysis and machine learning to discriminate the origin of *D. crepidatum*. Fifty batches of stem samples from Yunnan, Guangxi, and Guizhou, China, were analyzed after Savitzky-Golay smoothing, standard normal variate transformation, and first-derivative preprocessing. Principal component analysis (PCA) showed origin-related spectral variation, and a three-class partial least squares-discriminant analysis (PLS-DA) model achieved a mean cross-validated accuracy of 70.2% with a significant permutation-test result (*p* = 0.0020). Six machine learning algorithms, including KNN, CART, RF, NB, LDA, and ANN, were further compared using repeated nested cross-validation. KNN performed best, with an accuracy of 0.811 ± 0.029 and a macro F1-score of 0.813 ± 0.029, followed by RF (0.804 ± 0.038 and 0.805 ± 0.037, respectively). Key spectral variables were mainly located at 4231–4235 and 5523–5624 cm^−1^ corresponding mainly to C-H-dominated overtone or combination absorptions with possible C-O/O-H-related contributions from carbohydrates, polysaccharides, phenolics, flavonoids, and other organic constituents. These results demonstrate the feasibility of NIRS combined with machine learning for preliminary origin traceability of *D. crepidatum* and provide spectral clues for future investigation of origin-related chemical variation and quality discrimination.

## 1. Introduction

*Dendrobium crepidatum* Lindl. et Paxt. (*D. crepidatum*) is a medicinal *Dendrobium* species recorded in the Chinese Pharmacopeia, and its stems are commonly used as medicinal materials [1]. According to the Flora of China, *D. crepidatum* has traditionally been used for nourishing yin, benefiting the stomach, promoting fluid production, relieving irritability, and moistening the lungs to alleviate coughing. It has also been applied for conditions such as dry mouth and thirst, yin-deficiency-related fluid depletion, post-illness fever, blurred vision, pulmonary tuberculosis, and poor appetite [2].

The chemical characteristics and quality of medicinal plants may vary with geographic origin because ecological factors such as soil composition, climate, altitude, humidity, and light exposure can influence plant metabolism and secondary metabolite accumulation [3,4]. When medicinal materials from different regions are mixed, such variation may lead to inconsistent quality and affect the stability of practical application. Therefore, establishing a rapid and reliable method for geographic-origin traceability of *D. crepidatum* is important for batch management, market supervision, and quality-control screening.

Previous studies have used conventional identification, chemical analysis, chromatographic fingerprinting, and spectroscopic methods to evaluate the quality, authenticity, and origin-related variation in medicinal materials. For *Dendrobium* materials, HPLC fingerprints, HPLC-ESI-MS, and HPTLC analyses have been used to chemically differentiate *D. officinale* from *D. devonianum*, and chromatographic and mass spectrometric methods have been applied to characterize polysaccharides from *D. officinale* samples of different origins and production locations [5,6]. HPLC-based oligosaccharide spectrum-effect analysis and HPLC-DAD-ESI-QTOF-MS/MS have also been used to screen or identify quality-related markers in *Dendrobium* materials [7,8]. Similar chemical-composition-based strategies, including multi-element and metabolite profiling, FTIR combined with chemometric analysis, and HPLC fingerprinting, have been applied for geographical authentication, quality evaluation, and species differentiation of other medicinal materials [9,10]. Moreover, although chemical and chromatographic methods provide direct chemical evidence, they usually require extraction, solvent use, complex sample preparation, skilled operation, and partial sample consumption, which limits their application in rapid screening of large numbers of samples.

Near-infrared spectroscopy (NIRS) is a rapid and reagent-free analytical technique that captures overall spectral fingerprint information associated with organic functional groups in plant materials. The spectral acquisition itself causes little sample loss, although simple physical preparation such as drying and grinding may still be required to obtain homogeneous powder samples. Compared with conventional chemical analytical methods, NIRS does not require chemical extraction or solvent-consuming pretreatment during spectral measurement. When combined with chemometric or machine learning methods, NIRS can extract discriminative information from complex and highly overlapped spectral data [11,12,13,14,15]. For example, NIRS combined with PLS-DA and support vector-based models has been used to distinguish the geographic origin of *D. officinale* and predict multiple quality-related components using quantitative reference measurements [16]. NIRS and hyperspectral imaging combined with multivariate models have also been applied to classify agricultural products based on spectral and morphological information [17]. In addition, NIRS coupled with chemometric modeling has been reported for rapid authenticity identification and adulteration quantification of saffron using validated external prediction strategies [18]. These studies support the feasibility of NIRS combined with multivariate analysis and machine learning for origin discrimination and authenticity screening.

In this study, 50 batches of *D. crepidatum* samples were collected from Yunnan, Guangxi, and Guizhou, China, regions located within important distribution areas of *Dendrobium* resources in southern and southwestern China [19,20]. NIRS was combined with PCA, PLS-DA, and six machine learning algorithms, including KNN, CART, RF, NB, LDA, and ANN, to discriminate the geographic origins of *D. crepidatum* samples. In addition, spectral variables contributing to origin discrimination were evaluated to explore the possible spectral basis of regional differentiation. This study aimed to establish a rapid NIRS combined with machine learning workflow for preliminary geographic-origin screening of *D. crepidatum* and to provide spectral clues for future investigation of origin-related quality variation.

## 2. Materials and Methods

### 2.1. Instruments

Near-infrared spectrometer (MATRIX-F, Bruker , Ettlingen, Germany), Chinese herbal medicine crusher (800 A, Zhejiang Yongkang Red Sun Electromechanical Co., LTD., Yongkang, China), blast drying box (DHG-9140A, Shanghai Heng Scientific Instrument Co., LTD., Shanghai, China), electronic balance (AX224ZH/E, Ohaus Instrument Changzhou Co., LTD., Changzhou, China), weighing bottles (Beijing Wanjing Bomei Glass Products Co., LTD., Beijing, China), 60-mesh sieve (Wusi Textile Factory, Shangyu, China).

### 2.2. Materials

**Figure 1:**
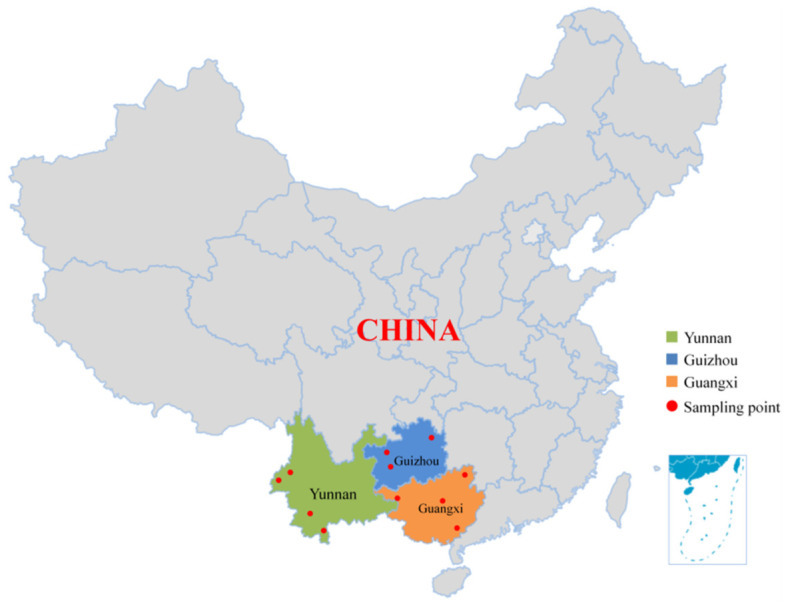
Sample collection map of *D. crepidatum*.

A total of 50 batches of fresh *D. crepidatum* stems were collected from Yunnan (*n* = 18), Guangxi (*n* = 16), and Guizhou (*n* = 16) , China (Figure 1 and Table 1). According to the recorded sample information, the Yunnan samples were collected from Ning’er, Jinggu, Dehong, Simao, Jinghong, Baoshan, Yingjiang, Menghai, Mengla, and Ruili; the Guangxi samples were collected from Laibin, Yulin, Guilin, and Baise; and the Guizhou samples were collected from Anshun, Bijie, and Tongren. Each sample was collected as an independent batch and processed separately. Some sample batches shared the same recorded sampling origin, such as M2 and M5 from Jinggu, M20–M22 from Yulin, M23–M25, M30, and M34 from Guilin, M35–M37, M47, and M48 from Anshun, and M38–M40, M42, M45, and M46 from Bijie. These samples were independently numbered and processed as separate batches.

The inclusion of samples from Yunnan, Guangxi, and Guizhou, which are located within important distribution areas of *D. crepidatum* and related *Dendrobium* resources, provided geographic coverage for this preliminary origin-discrimination study and supported a degree of regional representativeness within the sampled areas. Nevertheless, because the number of sample batches was limited and detailed information on harvest year and cultivation conditions was not systematically recorded for all samples, the dataset should be regarded as representative of the sampled regions rather than fully representative of all possible production conditions of *D. crepidatum*. All plant materials were taxonomically authenticated as *D. crepidatum* Lindl. et Paxt. by Professor Baozhong Duan of Dali University. Voucher specimens were deposited in the Herbarium of Traditional Chinese Medicine, Dali University. The fresh stems were cut into small pieces, dried to a constant weight at 60 °C, pulverized, and passed through a 60-mesh sieve. The resulting powders were stored in a desiccator until NIR spectral acquisition.

### 2.3. Experimental Methods

#### 2.3.1. NIR Spectral Acquisition

The dried *D. crepidatum* stems were pulverized and passed through a 60-mesh sieve. The powder was placed in a transparent glass weighing bottle to a depth of approximately 1 cm, and the surface was leveled before measurement. Near-infrared diffuse-reflectance spectra were acquired using the Bruker MATRIX-F spectrometer described above and controlled by OPUS v.7.8 software (Bruker, Ettlingen, Germany). Spectra were recorded over 12,000–4000 cm^−1^ at a resolution of 8 cm^−1^ using 32 co-added scans. A background spectrum was collected using the instrument reference before sample measurement. All measurements were performed at room temperature under consistent conditions. Each batch was repositioned and measured three times, and the mean spectrum was used for subsequent analysis.

#### 2.3.2. Spectral Preprocessing

Raw NIR spectra may contain high-frequency noise, scattering effects, and baseline variations that influence multivariate analysis. All spectra were therefore sequentially preprocessed using OPUS 7.8 software. Savitzky–Golay smoothing was first applied to reduce high-frequency noise while preserving spectral features. Standard normal variate transformation was subsequently used to reduce scattering-related variation, followed by first-derivative transformation to correct baseline shifts and enhance subtle spectral differences [21]. The same preprocessing sequence was applied to all samples. The raw and preprocessed NIR spectra were plotted using Python 3.12 with Matplotlib 3.10.9.

#### 2.3.3. PCA and PLS-DA Analysis

PCA was used to visualize the main spectral variation and sample distribution among the three geographic origins, and PLS-DA was used for supervised three-class discrimination. The geographic-origin variable was dummy-coded into three response columns representing Yunnan, Guangxi, and Guizhou. Both analyses used 803 preprocessed spectral variables within 7100–4000 cm^−1^. Before PCA, variables were mean-centered and scaled to unit variance. PLS-DA models with two to eight latent components were evaluated, and the final three-component model was selected using repeated stratified five-fold cross-validation with 20 repeats.

Model performance was assessed using accuracy, balanced accuracy, and an aggregated cross-validated confusion matrix based on held-out predictions from all repeated validation runs. Model significance was evaluated using 500 label permutations, and the *p* value was calculated with plus-one correction. PCA and PLS-DA analyses were performed using Python 3.12 with scikit-learn 1.7.2, and figures were generated using Matplotlib 3.10.9. The shaded regions in the score plots represent 95% data ellipses for visualizing within-group dispersion only.

#### 2.3.4. Machine Learning Model Development and Validation

Six machine learning algorithms, including KNN, CART, RF, NB, LDA, and ANN, were used to classify the geographic origins of *D. crepidatum* samples. All models were developed using the same dataset of 50 samples and 2074 preprocessed NIR spectral variables.

To ensure fair comparison, all algorithms were evaluated using identical outer training and validation partitions. A repeated nested cross-validation strategy was used, with 20 repetitions of five-fold stratified cross-validation in the outer loop for performance estimation and three-fold stratified cross-validation in the inner loop for feature selection and hyperparameter optimization. Model performance was evaluated using accuracy, balanced accuracy, macro-averaged precision, macro-averaged recall, and macro-averaged F1-score.

The key optimized settings were as follows: KNN used 200 selected variables and k = 1; CART used 50 selected variables and a maximum depth of 2; RF used all variables, 500 trees, and a maximum depth of 3; NB used 100 selected variables and a variance-smoothing value of 10^−11^; LDA used 100 selected variables and a shrinkage value of 0.5; and ANN used 100 selected variables and one hidden layer with 20 neurons. All machine learning analyses were performed using Python 3.12 with scikit-learn 1.7.2.

## 3. Results

### 3.1. NIR Spectral Characteristics of D. crepidatum from Different Origins

**Figure 2:**
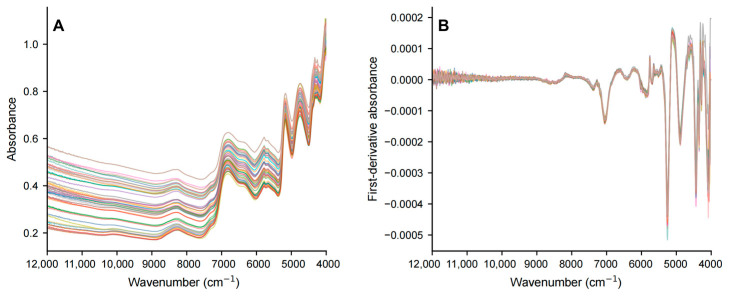
Raw and preprocessed NIR spectra of *D. crepidatum* samples. (**A**) Raw NIR spectra; (**B**) spectra after Savitzky–Golay smoothing, standard normal variate transformation, and first-derivative preprocessing. Different colors represent individual samples.

The original NIR spectra of *D. crepidatum* samples from different origins showed generally similar profiles and were difficult to distinguish directly (Figure 2A). After Savitzky–Golay smoothing, standard normal variate transformation, and first-derivative preprocessing, baseline drift and scattering effects were reduced, and subtle spectral features became clearer (Figure 2B).

According to general NIR band assignments, the 7100–6000 cm^−1^ region is mainly related to the first overtone of O-H stretching vibrations, which may reflect hydroxyl-containing constituents such as saccharides, polysaccharides, flavonoids, and phenolics. The 6000–5300 cm^−1^ region is associated with N-H, C-H, and O-H overtone absorptions, suggesting possible contributions from protein- or amino-acid-related components, carbohydrates, and flavonoids. The 5300–4900 cm^−1^ region corresponds mainly to O-H, N-H, C-H, and C=O combination bands, which may be related to saccharides, polysaccharides, and protein-related components. The 4900–4500 cm^−1^ region is associated with C-H, C=O, and O–H combination bands, indicating possible contributions from flavonoids, phenolics, and polysaccharides. The 4500–4000 cm^−1^ region is mainly assigned to C-H stretching/bending combinations and -CH_2_-related vibrations, which are often associated with polysaccharides, starch, proteins, and other carbohydrates [22,23]. Similar NIR regions have also been reported in plant- and food-related studies of geographical authentication and compositional discrimination [24,25,26,27,28].

Overall, these spectral regions provide a chemical basis for subsequent origin-discrimination analysis. However, because the spectra from different origins overlapped substantially, multivariate analysis and machine learning were further used to extract discriminative spectral information.

### 3.2. PCA and PLS-DA Analysis of Geographic Origin

**Figure 3:**
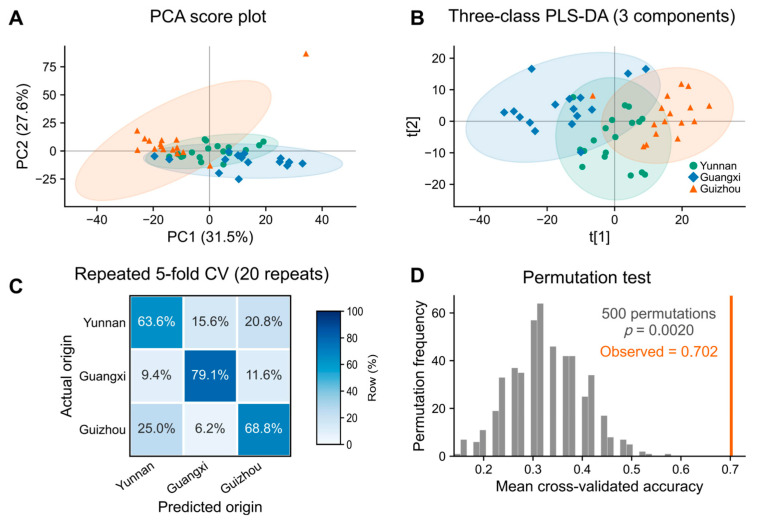
PCA and PLS-DA analysis of *D. crepidatum* samples from different origins. (**A**) PCA score plot; (**B**) three-class PLS-DA score plot; (**C**) aggregated cross-validated confusion matrix from repeated five-fold CV; (**D**) permutation test. Green circles, blue diamonds, and orange triangles indicate Yunnan, Guangxi, and Guizhou samples, respectively. Shaded ellipses indicate 95% data ellipses. The orange vertical line in panel D indicates the observed mean cross-validated accuracy.

PCA was first used to explore the natural distribution of *D. crepidatum* samples from different geographic origins. As shown in Figure 3A, PC1 and PC2 explained 31.5% and 27.6% of the total variance, respectively, accounting for 59.1% of the spectral variation. Samples from Yunnan, Guangxi, and Guizhou showed a certain clustering tendency, but substantial overlap remained among the three groups, indicating that PCA alone was insufficient for reliable origin discrimination. It should be noted that one Guizhou sample was located relatively far from the main Guizhou cluster in the PCA score plot. However, this pattern was not consistently observed for the same sample in the PLS-DA score plot. Therefore, this sample was interpreted as reflecting within-origin spectral heterogeneity rather than being removed as a confirmed outlier.

A supervised three-class PLS-DA model was then established to improve group separation. Compared with PCA, the PLS-DA score plot showed clearer separation among the three origins (Figure 3B). Guangxi samples were mainly distributed on the negative side of t[1], Guizhou samples on the positive side, and Yunnan samples between the two groups. However, partial overlap was still observed, suggesting that the origin-related spectral differences were moderate rather than completely separated.

Repeated five-fold cross-validation further evaluated the discriminative ability of the PLS-DA model. As shown in Figure 3C, the recall values for Yunnan, Guangxi, and Guizhou were 63.6%, 79.1%, and 68.8%, respectively, with an overall mean cross-validated accuracy of 70.2%. The permutation test with 500 random label permutations showed statistical significance (*p* = 0.0020), and the observed accuracy of 0.702 was higher than the permuted results (Figure 3D). These results indicate that PLS-DA captured meaningful origin-related spectral information, although further machine learning models were needed to improve classification performance.

### 3.3. Machine Learning-Based Classification of Geographic Origin

#### 3.3.1. Comparison of Optimized Machine Learning Models

Using the unified validation strategy described in Section 2.3.4, six optimized machine learning models were compared for geographic-origin classification of *D. crepidatum* samples. As shown in Table 2, KNN achieved the best overall performance, with the highest accuracy (0.811 ± 0.029) and macro F1-score (0.813 ± 0.029). RF ranked second, with comparable accuracy (0.804 ± 0.038) and macro F1-score (0.805 ± 0.037), indicating that both models effectively captured origin-related spectral differences. NB and ANN showed intermediate performance, with accuracies close to 0.79 and macro F1-scores around 0.79. Among them, NB had the smallest standard deviation, suggesting relatively stable performance across repeated validation runs. In contrast, CART and LDA showed lower classification performance, with accuracies below 0.75, indicating that these two models were less suitable for this dataset under the current modeling strategy.

Overall, the optimized models achieved moderate to good discrimination among *D. crepidatum* samples from different geographic origins. KNN provided the best balance of classification accuracy and macro F1-score, followed closely by RF.

#### 3.3.2. Confusion-Matrix Analysis of Geographic-Origin Classification

**Figure 4:**
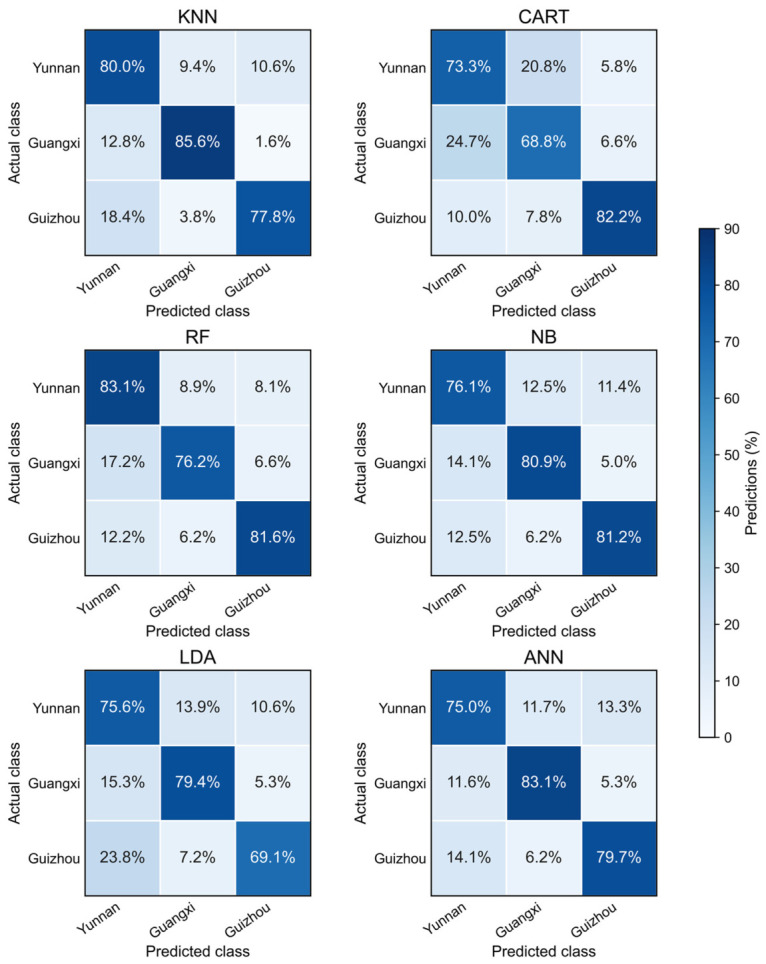
Cross-validated confusion matrices of the six optimized machine learning models for origin classification. Each matrix shows the aggregated prediction percentages from repeated nested cross-validation. Rows represent the actual origins, columns represent the predicted origins, and darker colors indicate higher prediction percentages.

The aggregated cross-validated confusion matrices further confirmed that the optimized machine learning models achieved meaningful geographic-origin discrimination and allowed class-specific classification patterns to be examined (Figure 4). In these matrices, diagonal cells represent correctly classified samples, whereas off-diagonal cells indicate misclassified samples, thereby providing class-specific information beyond the overall performance metrics shown in Table 2.

Overall, the confusion matrices supported the model ranking shown in Table 2. KNN and RF displayed stronger diagonal patterns, indicating higher correct classification proportions across the three origins. NB and ANN also showed acceptable classification patterns, whereas CART and LDA produced more off-diagonal errors. For the best-performing KNN model, the recall values for Yunnan, Guangxi, and Guizhou were 80.0%, 85.6%, and 77.8%, respectively, indicating that Guangxi samples were classified most accurately. The main misclassifications occurred between Yunnan and Guizhou, suggesting that their NIR spectral profiles were more similar than those involving Guangxi. Although similar misclassification patterns were observed across several models, KNN and RF showed fewer misclassified samples than CART and LDA. Together, these results indicate that the optimized machine learning models, especially KNN and RF, extracted useful discriminative information from the NIR spectra and provide insight into origin pairs with relatively similar spectral characteristics.

#### 3.3.3. Model-Relevant Spectral Variables for Origin Discrimination

**Figure 5:**
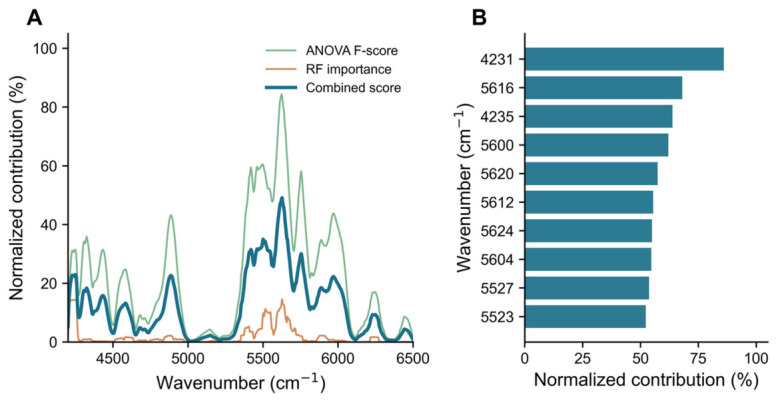
Spectral variables contributing to geographic-origin discrimination of *D. crepidatum*. (**A**) Normalized ANOVA F-score, RF importance, and combined contribution score in the 4200–6500 cm^−1^ region. (**B**) Top 10 wavenumbers ranked by the combined contribution score. The combined score was calculated from the normalized ANOVA F-score and RF importance. Higher values indicate greater contribution to geographic-origin discrimination.

To further interpret the spectral basis of geographic-origin classification, spectral variables contributing to origin discrimination were evaluated using normalized ANOVA F-scores, RF importance, and their combined contribution score (Figure 5). Although KNN achieved the highest overall classification performance, it is a distance-based method and does not provide direct variable-importance measures. Therefore, RF importance was used for spectral-variable interpretation because RF showed comparable classification performance and can estimate the contribution of individual wavenumbers to classification. The ANOVA F-score reflects the degree of inter-origin variation at each wavenumber, whereas RF importance reflects the contribution of each variable to classification in the random forest model [29,30]. The combined contribution score was calculated by integrating these two complementary indicators to identify spectral variables that showed inter-origin differences and contributed to model classification.

As shown in Figure 5A, the model-relevant signals were mainly concentrated in the 4200–6500 cm^−1^ region, with prominent contribution peaks around 4231–4235 cm^−1^ and 5523–5624 cm^−1^. The ANOVA F-score showed strong responses around 5600–5624 cm^−1^, indicating marked inter-origin spectral variation in this region. RF importance showed a pronounced contribution near 4231 cm^−1^, suggesting that this variable played an important role in model-based classification. The combined score therefore highlighted both the 4231–4235 cm^−1^ and 5523–5624 cm^−1^ regions as important spectral regions for origin discrimination. The top-ranked wavenumbers based on the combined contribution score were 4231, 5616, 4235, 5600, 5620, 5612, 5624, 5604, 5527, and 5523 cm^−1^ (Figure 5B).

According to general NIR band assignments, the 4231–4235 cm^−1^ region may be associated with C-H stretching/bending combination bands and C-O/O-H-related combination absorptions, whereas the 5523–5624 cm^−1^ region is mainly related to C-H first overtone absorptions and O-H/C-H-related spectral features [22,23]. These functional groups are widely present in carbohydrates, polysaccharides, flavonoids, phenolics, and other organic constituents. Therefore, the model-relevant variables may reflect differences in these chemical classes among *D. crepidatum* samples from different origins. However, further experimental validation using chromatographic or mass spectrometric analysis is still needed to confirm the specific chemical markers underlying these spectral associations.

## 4. Discussion

### 4.1. Chemical Interpretation of Origin-Related Spectral Differences

The present study showed that NIR spectroscopy combined with multivariate analysis and machine learning could distinguish *D. crepidatum* samples collected from Yunnan, Guangxi, and Guizhou. PCA provided an initial visualization of spectral variation, while PLS-DA further revealed spectral patterns associated with geographic origin. Although partial overlap remained among the three origins, this overlap is reasonable because samples from different regions belong to the same species and share similar major chemical constituents. Nevertheless, detectable spectral differences were still observed among origins. Although this study did not directly quantify specific metabolites, these spectral differences may reflect origin-related variation in the overall chemical profile of *D. crepidatum*, which could be influenced by environmental factors such as soil composition, climate, altitude, and light exposure [3,4].

After variable evaluation using ANOVA F-scores and RF importance, the spectral variables contributing most to geographic-origin discrimination were mainly located within the 4200–6500 cm^−1^ region, especially around 4231–4235 cm^−1^ and 5523–5624 cm^−1^. According to general NIR band assignments, these regions may be associated with C-H, C-O, and O-H-related overtone or combination absorptions [22,23]. These functional groups are commonly present in carbohydrates, polysaccharides, flavonoids, phenolics, and other organic constituents. Therefore, the model-relevant variables may be associated with variation in these chemical classes among samples from different origins. Previous phytochemical studies have confirmed that *D. crepidatum* and related *Dendrobium* species contain polysaccharides, flavonoids, alkaloids, phenolics, and other bioactive constituents, supporting the plausibility of this spectral interpretation [31,32,33,34]. However, these reports do not directly demonstrate that these constituents serve as confirmed markers for geographic-origin discrimination. Therefore, future studies should further combine chromatographic or mass spectrometric analysis for experimental validation to determine which specific compounds contribute to the observed spectral differences.

### 4.2. Model Performance, Algorithm Selection, and Validation

The multivariate and machine learning methods played complementary roles in geographic-origin discrimination. PCA provided an unsupervised overview of sample distribution, while PLS-DA improved supervised group separation. The cross-validated accuracy of PLS-DA was 70.2%, and the permutation test was significant (*p* = 0.0020), indicating that the observed separation was not caused by random class assignment. However, the moderate accuracy of PLS-DA also suggested that the origin-related spectral differences were not strong enough for complete separation using a single supervised projection model.

Six machine learning algorithms were therefore compared to determine which classification strategy was more suitable for extracting subtle origin-related information from the highly overlapping NIR spectra of *D. crepidatum*. These algorithms represent commonly used but methodologically different approaches for spectral classification: KNN evaluates local spectral similarity; CART and RF capture rule-based and nonlinear variable interactions; NB evaluates probabilistic class separation; LDA tests linear separability; and ANN explores more complex nonlinear spectral patterns. Because samples from different origins belong to the same species and share similar major constituents, their NIR spectra showed substantial overlap. Therefore, comparing multiple algorithms allowed origin-related spectral information to be evaluated from different modeling perspectives, consistent with previous NIR–machine learning classification studies [35,36,37,38,39,40].

Under the unified repeated nested cross-validation strategy, KNN achieved the highest average performance, followed closely by RF. NB and ANN showed comparable intermediate performance, whereas CART and LDA showed relatively lower classification performance. These differences may be related to the characteristics of NIR spectral data and the assumptions of each algorithm. NIR spectra are high-dimensional, highly collinear, and characterized by broad overlapping absorption bands. KNN may benefit from capturing local similarity after feature selection, while RF can handle nonlinear relationships and interactions among variables. In contrast, CART as a single decision tree may be sensitive to small changes in training data, and LDA assumes relatively linear class separation, which may not fully match the overlapping spectral distributions observed in this study. ANN can model nonlinear patterns, but its performance may be constrained by the limited sample size and high spectral dimensionality.

Importantly, all six machine learning models were evaluated using the same dataset, the same preprocessed spectral variables, and identical outer validation partitions. The outer cross-validation loop was used for performance estimation, whereas the inner loop was used for feature selection and hyperparameter optimization. This nested validation strategy improved the fairness of model comparison and reduced overly optimistic performance estimation during model selection [41,42,43]. Overall, the results suggest that a unified NIR–machine learning workflow, especially using KNN or RF, can provide a practical basis for rapid geographic-origin screening of *D. crepidatum*.

### 4.3. Practical Implications, Limitations, and Future Validation

These results demonstrate the feasibility of NIRS combined with machine learning for preliminary origin traceability of *D. crepidatum* and provide spectral clues for future investigation of origin-related chemical variation and quality discrimination. In production or market supervision, it could be used to rapidly screen batches from different declared origins, identify samples with spectral-origin profiles inconsistent with their declared sources, and support batch-level traceability management. Compared with conventional chromatographic or mass spectrometric methods, NIR spectroscopy is rapid, reagent-free, and causes little sample loss during spectral acquisition, which makes it suitable for high-throughput preliminary screening.

Although this study focused primarily on geographic-origin discrimination rather than direct quality evaluation, the origin-related NIR spectral differences may reflect variation in the overall chemical characteristics of *D. crepidatum*. Therefore, the proposed NIRS combined with machine learning strategy can provide spectral clues and a methodological reference for future quality discrimination studies [9,10,12,13,14,15]. However, the present results do not confirm specific quality markers or establish a direct correlation between NIR spectra and quality-related chemical components. Targeted chromatographic or mass spectrometric analyses are still required to confirm and quantify specific quality-related markers [5,7,8].

Several limitations should be acknowledged. First, the dataset included only 50 batches of samples, although they covered the major production provinces of Yunnan, Guangxi, and Guizhou [19,20]. Detailed metadata on harvest year, cultivation conditions, and production sources were not systematically recorded for all samples. Second, repeated nested cross-validation was used to reduce optimistic bias, but a completely independent external validation set was not available. Thus, the current performance mainly reflects discrimination within the present sampling framework and requires further validation using larger independent datasets [43]. Third, the chemical interpretation of discriminative spectral variables was based on NIR band assignments and model-derived variable evaluation rather than direct quantitative chemical evidence. Future studies should combine NIR spectroscopy with HPLC, LC-MS, or other validated reference methods to quantify candidate chemical indicators and confirm the specific chemical markers underlying the observed spectral differences [44].

Overall, the workflow combining NIRS with machine learning provides a feasible approach for rapid geographic-origin screening of *D. crepidatum* and a methodological reference for future quality-discrimination studies, although broader sample coverage and chemical validation remain necessary before wider practical application.

## 5. Conclusions

Near-infrared spectroscopy combined with multivariate analysis and machine learning enabled preliminary geographic-origin traceability of *Dendrobium* crepidatum samples from Yunnan, Guangxi, and Guizhou. PCA and three-class PLS-DA revealed origin-related spectral variation, while comparison of six machine learning algorithms under a repeated nested cross-validation framework showed that KNN achieved the best overall performance, followed closely by RF. The key spectral variables selected by model-based analysis were mainly located around 4231–4235 and 5523–5624 cm^−1^, suggesting that C–H-dominated overtone or combination absorptions, together with possible C–O/O–H-related contributions from carbohydrates, polysaccharides, phenolic compounds, flavonoids, and other organic constituents, may be associated with regional differentiation. These findings indicate that NIRS combined with machine learning can serve as a rapid screening tool for the origin traceability of *D. crepidatum* and provide spectral clues for future investigation of origin-related chemical variation and quality discrimination. Because these spectral clues have not yet been confirmed by independent chemical reference methods, expanding sample coverage and integrating reference chemical analysis will be necessary to refine the interpretation of the identified spectral variables and improve the applicability of this approach.

## Figures and Tables

**Table 1:** Information of samples.

No.	Origin	No.	Origin	No.	Origin
M1	Ning’er, Yunnan	M18	Ruili, Yunnan	M35	Anshun, Guizhou
M2	Jinggu, Yunnan	M19	Laibin, Guangxi	M36	Anshun, Guizhou
M3	Dehong, Yunnan	M20	Yulin, Guangxi	M37	Anshun Guizhou
M4	Simao, Yunnan	M21	Yulin, Guangxi	M38	Bijie, Guizhou
M5	Jinggu, Yunnan	M22	Yulin, Guangxi	M39	Bijie, Guizhou
M6	Jinghong, Yunnan	M23	Guilin, Guangxi	M40	Bijie, Guizhou
M7	Baoshan, Yunnan	M24	Guilin, Guangxi	M41	Tongren, Guizhou
M8	Dehong, Yunnan	M25	Guilin, Guangxi	M42	Bijie, Guizhou
M9	Dehong, Yunnan	M26	Baise, Guangxi	M43	Tongren, Guizhou
M10	Yingjiang, Yunnan	M27	Baise, Guangxi	M44	Tongren, Guizhou
M11	Yingjiang, Yunnan	M28	Laibin, Guangxi	M45	Bijie, Guizhou
M12	Jinghong, Yunnan	M29	Laibin, Guangxi	M46	Bijie, Guizhou
M13	Menghai, Yunnan	M30	Guilin, Guangxi	M47	Anshun, Guizhou
M14	Mengla, Yunnan	M31	Baise, Guangxi	M48	Anshun, Guizhou
M15	Mengla, Yunnan	M32	Laibin, Guangxi	M49	Tongren, Guizhou
M16	Ruili, Yunnan	M33	Baise, Guangxi	M50	Tongren, Guizhou
M17	Ruili, Yunnan	M34	Guilin, Guangxi		

**Table 2:** Cross-validated performance of machine learning models.

Model	Accuracy (Mean ± SD)	Balanced Accuracy	Macro Precision	Macro Recall	Macro F1 (Mean ± SD)
KNN	0.811 ± 0.029	0.811	0.817	0.811	0.813 ± 0.029
RF	0.804 ± 0.038	0.803	0.808	0.803	0.805 ± 0.037
NB	0.793 ± 0.019	0.794	0.794	0.794	0.794 ± 0.019
ANN	0.791 ± 0.052	0.793	0.792	0.793	0.792 ± 0.051
CART	0.747 ± 0.054	0.748	0.751	0.748	0.749 ± 0.054
LDA	0.747 ± 0.048	0.747	0.754	0.747	0.748 ± 0.047

## Data Availability

The original contributions presented in this study are included in the article. Further inquiries can be directed to the corresponding authors.
